# Do team and task performance improve after training situation awareness? A randomized controlled study of interprofessional intensive care teams

**DOI:** 10.1186/s13049-021-00878-2

**Published:** 2021-06-02

**Authors:** Karin Jonsson, Christine Brulin, Maria Härgestam, Marie Lindkvist, Magnus Hultin

**Affiliations:** 1 Department of Nursing and Department of Surgical and Perioperative Sciences, Anesthesiology and Critical Care Medicine, Umeå University, S-901 87 Umeå, Sweden; 2 Department of Nursing, Umeå University, S-901 87 Umeå, Sweden; 3 Department of Epidemiology and Global Health, Umeå University, S-901 87 Umeå, Sweden; 4 Department of Surgical and Perioperative Sciences, Anesthesiology and Critical Care Medicine, Umeå University, S-901 87 Umeå, Sweden

**Keywords:** Critical care, Interdisciplinary health team, Leadership, Patient safety, Simulation training, Situation awareness, Team performance

## Abstract

**Background:**

When working in complex environments with critically ill patients, team performance is influenced by situation awareness in teams. Moreover, improved situation awareness in the teams will probably improve team and task performance. The aim of this study is to evaluate an educational programme on situation awareness for interprofessional teams at the intensive care units using team and task performance as outcomes.

**Method:**

Twenty interprofessional teams from the northern part of Sweden participated in this randomized controlled intervention study conducted in situ in two intensive care units. The study was based on three cases (cases 0, 1 and 2) with patients in a critical situation. The intervention group (*n* = 11) participated in a two-hour educational programme in situation awareness, including theory, practice, and reflection, while the control group (*n* = 9) performed the training without education in situation awareness. The outcomes were team performance (TEAM instrument), task performance (ABCDE checklist) and situation awareness (Situation Awareness Global Assessment Technique (SAGAT)). Generalized estimating equation were used to analyse the changes from case 0 to case 2, and from case 1 to case 2.

**Results:**

Education in situation awareness in the intervention group improved TEAM leadership (*p* = 0.003), TEAM task management (*p* = 0.018) and TEAM total (*p* = 0.030) when comparing cases 1 and 2; these significant improvements were not found in the control group. No significant differences were observed in the SAGAT or the ABCDE checklist.

**Conclusions:**

This intervention study shows that a 2-h education in situation awareness improved parts of team performance in an acute care situation. Team leadership and task management improved in the intervention group, which may indicate that the one or several of the components in situation awareness (perception, comprehension and projection) were improved. However, in the present study this potential increase in situation awareness was not detected with SAGAT. Further research is needed to evaluate how educational programs can be used to increase situation awareness in interprofessional ICU teams and to establish which components that are essential in these programs.

**Trial registration:**

This randomized controlled trial was not registered as it does not report the results of health outcomes after a health care intervention on human participants.

**Supplementary Information:**

The online version contains supplementary material available at 10.1186/s13049-021-00878-2.

## Introduction

Preventable adverse events are common in intensive care units (ICU), both in Sweden [1] and globally [2]. Internationally, about 34% of patients in the ICU are affected by adverse events, and 60% of those events are avoidable [3]. Several studies have reported shortcomings in intensive care teamwork in respect to team leadership [4] and patient outcomes [5]. Fewer studies have focused on ICU teams’ situation awareness (SA) in order to improve team and task performance [6]. SA is described as being able to perceive what is going on (perception), to assess and interpret the situation (comprehension) and to project into the future (projection) [7].

SA is important in order to make the right decision at the right time. Attention to and knowledge of SA increase safety and reduce errors in high-risk organizations [8]; recently, Brennan [9] reported that such knowledge has improved patient safety in healthcare systems. It is clear that teamwork in the ICU depends on individuals’ coordinated actions and understanding of tasks, including collective cognitive structures such as shared mental models to optimize care [10]. Similarity, SA in teams has been shown to influence mental models and, consequently, decision-making processes [11, 12]. However, the role of SA in teams working in the ICU has been sparsely investigated and requires further examination.

Shared SA is a precursor of decision-making and is necessary for team coordination and performance [13]. Specific and targeted educational programmes to increase SA have been developed for aviation, the military [11], the police [14] and operating room personnel [15]. Education in SA has been suggested to be effective for the detection of patient deterioration [16]. However, a recent systematic review on educational interventions to enhance SA in healthcare showed that only two of the 39 included studies had an ICU context and neither was randomized; furthermore, only four of these studies had a specific focus on SA in the interventions [17]. Thus, specific randomized intervention studies evaluating SA education in respect to non-technical skill (NTS) outcomes such as team and task performance within healthcare are still lacking.

Team and task performance in well-functioning teams are essential for high-quality care in critical care settings [2]. Trauma teams with leaders that can communicate SA and encourage backup behaviours are able to perform better teamwork [18]. Team performance, as measured by leadership, team work and task management [19] in relation to SA, has been sparsely investigated. In a recent review, Schulz et al. [20] pointed out the importance of SA in the decision-making process, which affects team performance [21]. However, less attention has been paid to the association between SA and task performance.

In sum, SA plays an important role in improving the efficiency of intensive care teams. We hypothesized that a brief educational programme for intensive care teams with a focus on SA in a critical situation would improve teamwork outcomes such as: (1) SA; (2) overall team performance including leadership, teamwork and task management; and (3) task performance. Thus, this randomized intervention study aimed to evaluate an educational programme in SA for intensive care teams using team and task performance as outcomes.

## Methods

This randomized controlled intervention study evaluated an ICU team training educational programme that focused on SA with in situ simulations, using questionnaires and video-recorded observations. The study was performed in two hospitals (H1 and H2) in the northern part of Sweden.

### Participants

Enrolled nurses (ENs), critical care nurses (CCRNs) and physicians (MDs) working at two different ICU during the spring of 2017 were invited to participate in this study. A total of 215 individuals (H1 *n* = 104 vs. H2 *n* = 111) were employed at the studied ICUs. At the time of this study, 176 individuals were active in the work at the ICU and were thus invited to participate.

**Fig. 1:**
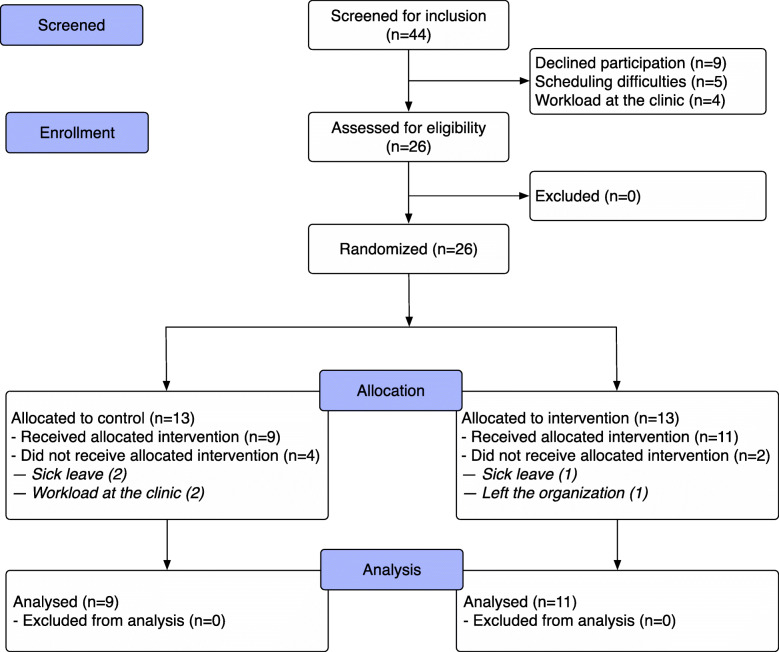
CONSORT diagram at the team level

After the baseline session, referred to herein as session one (case 0), all personnel (*n* = 105, 26 teams) from the baseline session were invited to participate in the intervention. Before organizing the follow-up – that is, session two (cases 1 and case 2) – the teams were stratified by hospital; thereafter, the participants at each hospital were randomized by drawing of lots into a control or intervention group (Fig. 1). In all, 75 of the 105 invited individuals participated in the follow-up, with nine teams in the control group and 11 teams in the intervention group. The reasons given for dropping out of the follow-up were mainly scheduling difficulties and high workload at the clinics. The follow-up included 20 teams. Most participants were female (59 of 75), and the participants’ ages ranged from 28 to 64 years (Table 1). Thirteen teams consisted of four individuals and seven teams consisted of three or five individuals. All teams included at least one of each of the listed professionals, except for one team that lacked an EN.

**Table 1:** Background characteristics of participants and teams in session two. Age and prior work experience are expressed as mean (standard deviation). Remaining variables are binary. At the team level, the proportion of participants in the teams belonging to the indicator category are shown as mean (standard deviation)

Characteristics	Individual level			Team level		
	*m (sd)*			*m (sd)*		
	Control (*n* = 32)	Intervention (*n* = 43)	*p* (*t*-test)	Control (*n* = 9)	Intervention (*n* = 11)	*p* (*t*-test)
Age	45.8 (9.7)	45.9 (9.5)	.978	46.2 (5.86)	45.9 (5.82)	.908
Prior work experience	16.6 (9.9)	18.3 (10.1)	.476	17.0 (6.17)	18.4 (5.35)	.593
	*n (%)*			*m (sd)*		
	Control	Intervention	*p* (*X*^*2*^)	Control	Intervention	*p* (*t*-test)
Gender female	23 (72)	36 (84)	.216	0.72 (0.13)	0.83 (0.21)	.209
Gender male	9 (28)	7 (16)	–	0.28 (0.13)	0.17 (0.20)	.209
Hospital 1	20 (63)	19 (44)	.116	0.66 (0.50)	0.45 (0.52)	.369
Hospital 2	12 (37)	24 (56)	–	0.33 (0.50)	0.54 (0.52)	.369
Profession EN	10 (31)	12 (28)	.879	–	–	–
Profession CCRN	13 (41)	20 (46)	–	–	–	–
Profession MD	9 (28)	11 (26)	–	–	–	–
MD at specialist level	7 (78)	8 (73)	.795	0.78 (0.44)	0.73 (0.47)	.808
Former courses	18 (56)	33 (77)	.060	0.55 (0.26)	0.76 (0.18)	.041*
Former experience CRM	15 (47)	23 (54)	.571	0.45 (0.30)	0.53 (0.23)	.452
Former experience debriefing with video	14 (44)	18 (42)	.870	0.42 (0.24)	0.41 (0.20)	.964
Former education leadership	5 (16)	11 (26)	.298	0.16 (0.15)	0.27 (0.22)	.201
Former education communication	8 (25)	12 (28)	.778	0.24 (0.27)	0.28 (0.18)	.730
Former team training with video	21 (66)	27 (63)	.800	0.63 (0.27)	0.62 (0.31)	.968
Former team training no video	26 (81)	37 (86)	.575	0.79 (0.29)	0.88 (0.25)	.437
Positive attitude re value of team training	30 (94)	37 (86)	.285	0.94 (0.13)	0.85 (0.18)	.274
Perception of daily frequency of team work	29 (91)	41 (95)	.417	0.91 (0.19)	0.95 (0.10)	.483
Perception of good quality of team work	30 (94)	41 (95)	.630	0.94 (0.11)	0.92 (0.15)	.661

Information about the study was provided to the participants in both written and verbal form at site meetings and by mail. Although participation in this study was voluntary, the team training sessions were mandatory for all employees. CONSORT reporting of trials was used. Consent for the project was obtained from the clinic management, and the study was approved by the Regional Ethical Review Board in Northern Sweden (April 7, 2016, Decision No. 2016–54-31 M). Informed consent was obtained from all participants.

### Setting

As mentioned earlier, the setting was in situ team training at two ICUs in two hospitals. The team training was performed using a patient simulator, Laerdal SimMan (3G). For standardization of the sessions, the patient simulator was pre-programmed with timed interventions for similar responses to performed actions. All other equipment was the usual equipment from the clinical environment. Two high-definition cameras were mounted to record the scenarios.

### Procedure and team training for the intervention and control group

**Fig. 2:**
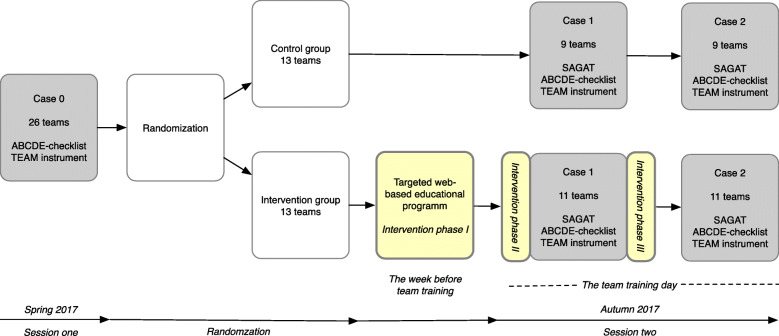
Flow chart of the progress of the study showing the simulation sessions (grey boxes), randomization (white boxes) and interventions (yellow boxes)

Session one was performed over 11 weeks during the spring of 2017. The same teams were then scheduled for session two, the follow-up training session including the intervention, during 8 weeks in autumn 2017 (Fig. 2). The baseline data from session one were analysed separately and recently published [21]. In session two, the participants trained using two different cases (case 1 and case 2). All three scenarios were designed to last 15–20 min and were first tested on physicians and CCRNs/ registered nurse anesthetists not involved in the study.

Six instructors at each hospital facilitated the training sessions, two at a time. Between session one and session two, the instructors (*n* = 6) in the intervention group received a one-day educational session including SA and theories of didactics and experiential learning [22]. Before the start of both sessions one and session two, all participants received a short pre-briefing that focused on the goals of the training and were asked to act as if they were experiencing an ordinary day at work. The participants were also introduced to the training room, facilities and patient simulator. The patients in the cases were in directly life-threatening situations. Case 0 suffered from several airway problems, case 1 suffered from obstructive pulmonary disease and case 2 was experiencing severe septic shock.

### Training for the intervention group

In addition to the simulation cases with reflective debriefings, which were the same as those provided in the control group, the intervention group were given a web-based educational programme (*phase I*), and two reflection sessions that focused on SA (*phase II* and *phase III*) (Fig. 2 and Supplementary Table 1).

#### Phase I

One week before session two, the participants in the intervention group went through a one-hour web-based educational programme. The programme consisted of four brief video lectures, three reflective questions to process the content, and five multiple-choice questions at the end. The theoretical parts focused on how to achieve SA. The programme also included theory on interprofessional teamwork, patient safety, NTSs and reflective learning. The learning objectives were focused on: (1) human factors and their significance for clinical practice [23, 24]; (2) theories on how team members can optimize their abilities and identify their own strengths and weaknesses [25–27], (3) descriptions for understanding SA [7]; and (4) theories on how to use SA knowledge in clinical practice [6, 28]. Each participant had an individual log in, which permitted personalized reminders to complete the programme.

Illustrations and a process description of SA were used with permission from *Situational Awareness and Patient Safety*, a resource developed by the Canadian Medical Protective Association and the Royal College of Physicians and Surgeons of Canada [28].

#### Phase II

Immediately before session two, the intervention group had a one-hour instructor-facilitated reflective discussion on the key concepts from *phase I*.

#### Phase III

After case 1 in session two, the participants had a reflective debriefing session [29] in a nearby room together with the instructors. The different phases of reflection – namely, reaction, analysis, summary and application – were discussed in order to facilitate experiential learning [22]. For the intervention group, the focus was on SA and how to turn theory into practice.

Finally, the participants were introduced to case 2 and were urged to apply their knowledge of SA and team skills.

### Training for the control group

For the control group, the web educational programme (*phase I*) and the two reflection sessions (*phases II* and *III* in the intervention) were not included in the training. However, after case 1, in session two, the control group had a debriefing session that included the same didactic as that provided to intervention group, except that it did not focus on SA (Table 1 and Supplementary Table 1).

### Data collection

**Fig. 3:**
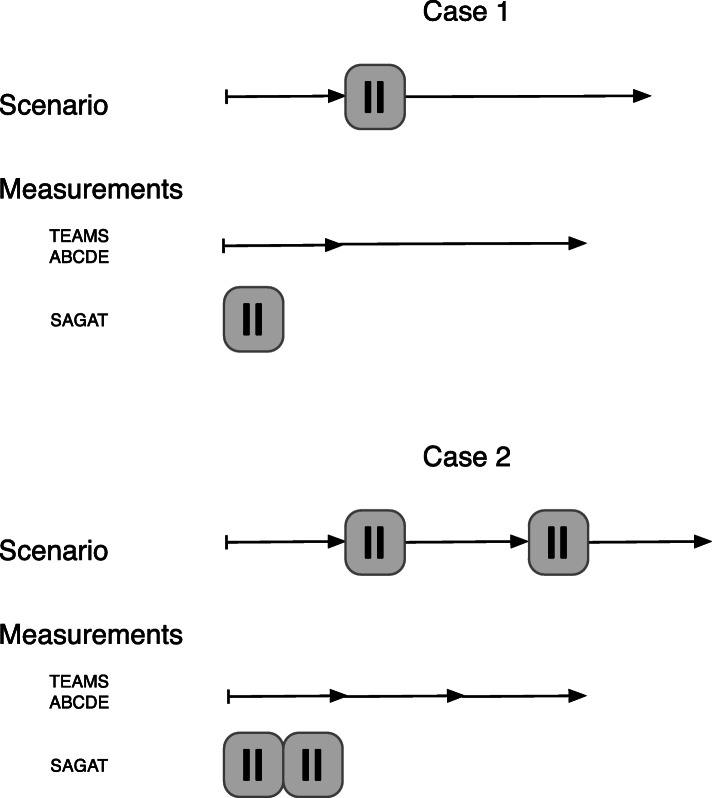
Flow chart describing when measurements were used during cases 1 and 2

In order to evaluate the SA educational programme, questionnaires and video recordings of cases 0, 1 and 2 were used. The questionnaires included background characteristics and the Situation awareness global assessment technique (SAGAT) (cases 1 and 2), the TEAM instrument and the ABCDE checklist (cases 0, 1 and 2; Figs. 2 and 3).

### SAGAT

Within healthcare, the SAGAT is the most commonly used method to measure SA [30, 31]. Its feasibility within a Swedish context has been tested by our research group [32]. SAGAT was designed to measure three levels of SA: (1) perception, (2) comprehension, and (3) projection. SAGAT uses goal-directed task analysis, as initially described by Endsley [11], to develop SA items for each level. Major goals for the session are identified by expert and the questions are thereafter constructed to the specific SA recruitments. (Supplementary Table 2). The technique involves a “freezing” activity at set points during the simulation; during the freezing activity, the SAGAT questionnaires should be distributed to the participants [11]. In case 1, a questionnaire with 13 items were distributed to the participants around 5 mins into the scenario. In case 2, 26 items were split into two different questionnaires, with the first “freeze” including 14 items and the second freeze including 12 items. The first freeze occurred 5 mins into the scenario and the second freeze took place 5 mins after the scenario resumed. During the freeze times, the patient monitor was switched off. The items were either free text or multiple-choice items (Supplementary Table 2 and 3). The answers given by the participants were classified as either incorrect (0) or correct [1] by two of the authors (KJ, MHu). Individual SAGAT scores were calculated as the sum of the items and then team SAGAT (TSAGAT) was calculated as the mean score in each team, giving a TSAGAT between 0.00–13.00 in case 1 and 0.00–26.00 in case 2.

### The TEAM instrument

The TEAM instrument was used to measure team performance [33]. The TEAM instrument includes three subscales: *leadership* (2 items), *teamwork* (7 items), and *task management* (2 items), and includes one item for the assessment of *overall* performance. All items except for the overall item were scored on a scale from 0 to 4 (Never/Hardly ever = 0, Seldom = 1, About as often as not = 2, Often = 3, Always/Nearly always = 4), and *overall* was scored from 1 to 10. Based on items in the respective subscale, mean scores were calculated for *leadership*, *teamwork*, and *task* management and given values between 0.00–4.00. In addition, based on all the items in the TEAM instrument (*n* = 11), a mean score was calculated (*total*) and given values between 0.00–4.00. Thus, the *total* score included all items measuring *leadership*, *teamwork*, and *task* management. The overall team performance item only consisted of one question and was therefore handled as a single overall item (*overall*) [33].

### ABCDE checklist

The ABCDE checklist was developed out of the ATLS protocol [34], and the checklist has been tested for feasibility and interrater reliability by our research group [32]. In this study, the ABCDE checklist [32] was used to measure task performance in terms of how the teams managed the airway (one item), breathing (five item), circulation (two items), disability (one item) and exposure (one item), for a total of 10 items. Each item was scored from 0 to 3 (Not initiated = 0, Performed partly = 1, Performed completely = 2, Performed consistently during the whole simulation = 3, Not applicable = NA). Based on all the items in the scale, a mean score was calculated, and a score from 0.00 to 3.00 was given. One item from breathing assessments was deleted in case 1.

Two of the authors (MHu and MHä) independently reviewed the recorded videos and scored the TEAM instrument and the ABCDE checklists, blinded to the control and intervention group.

### Statistical analyses

Statistical analysis was performed using IBM SPSS Statistics for Windows v.25. Background characteristics were described at the individual and team level (Table 1). At the individual level, age and prior work experience were presented as means and standard deviation. At the team level, the variables were aggregated, giving mean values for age and prior work experience. The remaining variables were binary, and mean values were used to show the proportion of participants in the teams belonging to the indicator category. For analyses of differences in background characteristics, independent sample *t*-tests or the Chi-square test were used, as indicated in Table 1.

According to the aim of this study, each team was defined as a unit. The means of the individuals in each team for each scale (TSAGAT, ABCDE and TEAM) were used as dependent variables. Normality for each dependent variable was checked with skewness.

The effect of the intervention on education was analysed with the generalized estimating equation (GEE) [35], assuming a scale linear model with an independent correlation matrix. The models accounted for the testing between the intervention and control group, the time (case 0, case 1 and case 2) and changes over time between the intervention and control group. We used GEE models to analyse the changes from case 0 to case 2, and from case 1 to case 2, respectively. Significance was set at *p* < 0.05.

### Study sample

Calculation of statistical power for the TEAM instrument was performed to determine the number of participants using G*Power [36]. From previously published data (Table 3 in Cooper et al. [33]) a mean TEAM total of 2.49 with a standard deviation of 0.91 was calculated. With a power of 80% at the 0.05 significance level, a total of 22 teams would be needed to detect an increase in the mean of 1.0.

## Results

In this randomized study, no significant differences were found in background characteristics between the intervention and control group regarding education, former crisis resource management (CRM) knowledge, former team training or proportion of MDs being specialists. The only significant difference between the groups was former courses at a team level (*p* = 0.041; Table 1).

After education in SA, the TEAM subscales *leadership* and *task management* as well as the *total* score improved significantly in the intervention group from case 1 to case 2, as compared with the control group (*p* = 0.003, *p* = 0.018 and *p* = 0.030; Table 2). More specifically, in the intervention group, the mean value for the subscale *leadership* increased from case 1 to case 2 (2.67, SE .11 vs. 3.17, SE .10), while there was no corresponding increase in the control group (2.50, SE .12 vs. 2.59, SE .11). The mean value for the subscale *task management* increased between case 1 and case 2 for the intervention group (2.50, SE .11 vs. 3.12, SE .07) and the corresponding increase for the control group was lower (2.60, SE .08 vs. 2.85, SE.14). The mean value for *total* increased between case 1 and case 2 for the intervention group (2.50, SE .08 vs. 2.91, SE .08), while the corresponding increase for the control group was somewhat lower (2.47, SE.08 vs. 2.70, SE .10; Table 2).

**Table 2:** Estimated mean score and changes between the intervention and control group on outcomes for case 0 vs case 2, and for case 1 vs case 2, respectively

	Intervention group mean (SE)			Control groups mean (SE)			Differences in mean change between intervention and control	
	case 0 (*n* = 13)	case 1 (*n* = 11)	case 2 (*n* = 11)	case 0 (*n* = 13)	case 1 (*n* = 9)	case 2 (*n* = 9)	case 0 versus case 2	case 1 versus case 2
**TEAM instrument**								
*Leadership* (score 0.00–4.00) ^a^	2.77 (.11)	2.67 (.11)	3.17 (.10)	2.48 (.17)	2.50 (.12)	2.59 (.11)	mean change = .30 SE = 0.20 *p* = .149	mean change = .42 SE = 0.14 ***p*** **= .003**
*Teamwork* (score 0.00–4.00) ^a^	2.58 (.09)	2.45 (.08)	2.78 (.10)	2.50 (.14)	2.43 (.10)	2.70 (.10)	mean change = .01 SE = 0.13 *p* = .962	mean change = .06 SE = 0.08 *p* = .508
*Task management* (score 0.00–4.00)^a^	2.73 (.13)	2.50 (.11)	3.12 (.07)	2.71 (.15)	2.60 (.08)	2.85 (.14)	mean change = .24 SE = .17 *p* = .157	mean change = .36 SE = .15 ***p*** **= .018**
*Overall* (score 0.00–10.00) ^a^	6.92 (.24)	6.55 (.19)	7.87 (.16)	6.77 (.28)	6.61 (.26)	7.61 (.19)	mean change = .10 SE = .36 *p* =. 775	mean change = .32 SE = .23 *p* = .174
*Total* (score 0.00–4.00) ^a^	2.64 (.09)	2.50 (.08)	2.91 (.08)	2.53 (.14)	2.47 (.08)	2.70 (.10)	mean change = .10 SE = .13 *p* = .434	mean change = .18 SE = .08 ***p*** **= .030**
**ABCDE-checklist** (score 0.00–3.00) ^a^ 9 item case 1 10 item case 2	2.14 (.06)	2.21 (.05)	2.52 (.03)	2.11 (.06)	2.09 (.08)	2.48 (.07)	mean change = .01 SE = .10 *p* = .897	mean change = − .084 SE = .12 *p* = .492
**TSAGAT** (score 0.00–1.00) ^b^		.45 (.03)	.55 (.03)		.48 (.03)	.49 (.02)		mean change = .08 SE = .05 *p* = 0.062

^a^ higher score indicates a higher degree of performance

^b^ higher score indicates a higher SA

No significant differences were found between the intervention and control group in the TEAM subscales *teamwork* and *overall*, the TSAGAT or the ABCDE checklist (Table 2).

## Discussion

This randomized controlled intervention study demonstrates improvements in team performance for intensive care teams after a 2-h, three-phase educational intervention with a focus on SA. Both the intervention and the control group participated in three full-scale simulation scenario training cases with debriefings. The intervention group increased its TEAM *total* score from case 1 to case 2, mainly due to improvements in the subscales *leadership* and *task management.*

This study builds on prior research indicating that team training improved team performance [37] and that well-functioning task work was found to be essential for efficient teamwork [38]. Frequent sharing of mental models and frequent back up team leader behaviour correlated to better team performance [18]. SA has been found to be essential when developing mental models [39], and simulation based education has been shown to be an effective learning modality to enhance SA [17]. Moreover, in a qualitative study ICU nurses described that simulation based team training will prepare ICU nurses in taking care of severely ill patients and achieve a more common understanding in teamwork towards patient safety [40]. Thus, the intervention in this study was designed to facilitate a process where the multidisciplinary team, consisting of a mixture of competences and professions, work together to enhance their performance. In the present study we found an effect of the intervention on team performance but not did not find an effect on SA as measured by SAGAT. In a randomized trial, Hänsel et al. [41] studied the effect of simulation training compared to traditional CRM course on team performance and SA. In the group that received simulator training, SA (as measured by SAGAT) increased compared to the group participating in a traditional CRM course. A systematic review comparing the effectiveness of educational interventions on healthcare professionals’ SA, showed that simulation based education as the most effective educational modality to enhance SA. However, of 39 included studies, only four had educational interventions specifically focusing on SA. In the other included studies, SA was only a small part of the educational intervention [17]. Thus, further research on the effects of educational interventions specifically focusing on SA is needed.

It is not a common practice in the literature to use the SAGAT to measure SA in ICU scenarios [42]. Cooper et al. [43] identified 14 studies measuring SA in emergency settings; of these, four used the SAGAT, but not in an ICU context. Recently, Coolen et al. [44] showed a correlation between the SAGAT, team task prioritization, problem solving and rapid diagnosis within paediatric acute care teams. In the present study, training in SA improved the professionals’ abilities to manage critical situations, as measured with the TEAM instrument; however, the difference in mean change in the TSAGAT between the control and intervention group did not reach a significant level.

The limitations in this study require consideration. The SAGAT is measured by asking participants to answer questionnaires while the running scenario has been paused. Like all tests, SAGAT sensitivity is affected by small samples and few items [42]. It is possible that the relatively low number of items (13 in case 1 and 26 in case 2) might explain the lack of a significant difference in the SAGAT for the intervention group, as a greater number of items in the questionnaires might have increased the sensitivity of the SAGAT measurements.

A two-hour intervention might be considered as small compared to the effect size postulated in the power analysis. When planning the study, our assumption was that the educational intervention would have the potential to substantially increase TEAM performance. This brief, but systematic, training in enhancing SA did result in significant improvements in TEAM total, and in the subscales leadership and task management. Most likely, however, more teams would have been beneficial to enhance the power of the study. For example, the difference in SAGAT between intervention and control was borderline to becoming significant (*p*-value 0.06), which might be a type II error.

Running full-scale in situ simulations in a critical care ward was challenging since resources might be dynamically reallocated from the educational programme to meet urgent clinical needs. It is well established that efficient team training requires effort [45, 46], and there is an association between learning outcomes and hours of training [47]. Repeated training sessions are needed for the retention of team training knowledge [45], as well as to achieve positive effects through CRM training [48].

This randomized controlled intervention study demonstrates that a small educational intervention, consisting of a web module with follow-up group discussion in preparation for an in situ simulation session, may increase the positive effects of simulation based education. As Parush [49] describes, SA is a tacit viable concept, highly relevant in the clinical context since specific SA education effect task as well as team performance and to achieve high SA is a trainable skill for both novices and well experienced staff. Focusing on obtaining SA – that is, the process of how the team as a whole notice the situation (perception), understands how to interpret the situation (comprehension) and realizes how the situation is about to develop (projection) – was found to increase team performance. It is still too early to draw definitive conclusions based available studies regarding which educational intervention that is most effective for optimizing team SA. Simulation as an educational modality is beginning to be evaluated favourably for healthcare [17], but still details as length of the intervention, repetitions, and team sizes remains elusive.

## Conclusion

This study stresses the importance of education in SA in intensive care teams in order to improve team and task performance. SA influence mental models and is a precursor in decision-making and is therefore necessary for team coordination and performance. This study found improvements in the TEAM subscales leadership and task management after a 2-h educational intervention focusing on SA. The predicted increase in SA was not detectable, at least not with SAGAT. Randomized controlled educational in situ simulation studies are resource intensive but are needed to achieve evidence-based education. Further research is required to evaluate whether enhancing team SA can effectively improve team performance.

## Supplementary Information

**Additional file 1: Supplementary Table 1.** Overview of differences in the educational programme between the control and intervention group. **Supplementary Table 2.** Goal directed task analysis document case 2. **Supplementary Table 3.** SAGAT questionnaire case 2, freeze one and two.

## Data Availability

The dataset used and analyzed during the current study are available from the corresponding author on reasonable request.

## Abbreviations

CCRN: Critical care nurses
EN: Enrolled nurses
GEE: Generalized estimating equation
ICU: Intensive care units
MD: Physicians
NTS: Non-technical skill
SA: Situation awareness
SAGAT: Situation awareness global assessment technique
TSAGAT: Team situation awareness global assessment technique
