# Supplementary material for: Do team and task performance improve after training situation awareness? A randomized controlled study of interprofessional intensive care teams
Source: Scand J Trauma Resusc Emerg Med. 2021 Jun 2;29:73. doi: 10.1186/s13049-021-00878-2 (PMC8170734; doi:10.1186/s13049-021-00878-2)
Supplement: Supplementary file 1 — Additional file 1: Supplementary Table 1. Overview of differences in the educational programme between the control and intervention group. Supplementary Table 2. Goal directed task analysis document case 2. Supplementary Table 3. SAGAT questionnaire case 2, freeze one and two. [file 13049_2021_878_MOESM1_ESM.docx]

**Supplementary material to manuscript**:

STRE-D-20-00432 entitled "Do team and task performance improve after training situation awareness? A randomized controlled study of interprofessional intensive care teams."

**Supplementary Table 1** Overview of differences in the educational programme between the control and intervention group.

| Subject | Time | Control | Intervention |
| --- | --- | --- | --- |
| Team training session one, case 0, including debriefing session through different phases of reflection: reaction, analysis, summary and application. | 2 h | Yes | Yes |
| ***Phase I*:** Web education for participants; theory with a focus on SA, NTS and team work skills. Reflective questions and quiz. | 1 h | No | Yes |
| ***Phase II*:** Reflection with a focus on previous education, questions and quiz. | 1 h | No | Yes |
| Introduction to the simulation procedures and learning objectives. | 15 min | Yes | Yes |
| Team training session two, case 1. | 30 min | Yes | Yes |
| ***Phase III*:** Debriefing session after case 1 through the different phases of reflection: reaction, analysis, summary and application. Focus during the debriefing session on how to increase SA within the team. | 30 min | No | Yes |
| Debriefing session after case 1 through the different phases of reflection: reaction, analysis, summary and application. | 30 min | Yes | No |
| Team training session two, case 2. | 30 min | Yes | Yes |
| Debriefing session after case 2. | 15 min | Yes | Yes |

**Supplementary Table 2 Goal directed task analysis document case 2**

| Huvudmål;  Stabilisering av patient med septisk chock | Delmål;  Bedöma och säkra luftväg, stabilisera respiration och cirkulation, nyttjande av resurser och systematiskt omhändertagande, följa SOP |
| --- | --- |
| Beslut; Stabilisering av respiration och cirkulation genom intubation och inotropi, AB |  |
| Krav på lägesbild;  Identifiera kliniska tecken på akut sepsis;  -hypoventilation  -hypotension  -sepsismekanismer  -beslut om fasciotomi r/t nekrotiserande fascit  Medvetenhet om fysiologiska grunder till sepsis;  - bakterietoxiner aktiverar försvarsystem, cytokinfrisläpp  -aktivering av koagulation  -dilaterat artärsystem, hypotension  Medvetenhet om naturliga följder till sepsis;  -akut svikt av respiration och cirkulation  -hypoperfusion  -hypotension  -organdysfunktion |  |
| Sökfrågor (essentiella frågor) | **Level 1 Perception;**   - Vad är patientens AF - Vad är fynden från bedömning av B - Vad är patientens SBT - Vad har patienten för HF - Är patienten adekvat utrustad (antal PVK) - Viktiga observationer (patientens halsband) - Tidigare sjukdomar - Vad är fynden från bedömning av C - Finns adekvat utrustning (fungerande sug) |
|  | **Level 2 Förståelse;**   - Är patienten tillräckligt syresatt - Orsaker till fysiologiska svar/symtombild - Fynd och orsak till bedömning av C |
|  | **Level 3 Förutsäga;**   - Vilka undersökningar kommer behöva utföras - Vad tror du kommer att hända med patientens HF närmaste 10 minuterna? - Vad tror du kommer att hända med patientens BT närmaste 10 minuterna? - Vad tror du kommer att hända med patientens Sat närmaste 10 minuterna? - Vad tror du kommer att hända med patientens AF närmaste 10 minuterna? - Vad tror du kommer att hända med patientens medvetande närmaste 10 minuterna? - Vad blir nästa steg i behandling |

**Supplementary Table 3 SAGAT questionnaire case 2, freeze one and two.**

| **Level** | **Fråga** | **Svar** |
| --- | --- | --- |
| 1 | Vad är patientens AF? | <6 □ 6-9 □ 10-15 □ 16-20□ 21-29□ 30-35□ >35□ Vet ej □ |
| 1 | Vad är fyndet från bedömningen av B på höger respektive vänster lunga? | Rena andningsljud hö □ vä □  Rhonki hö □ vä □  Rassel hö □ vä □  Stridor hö □ vä □  Dämpade hö □ vä □  Vet ej □ |
| 2 | Är patienten tillräckligt syresatt? | Ja □ Nej □ Vet ej □ |
| 1 | Vad är patientens systoliska BT? | <70□ 70-79□ 80-89□ 90-109□ 110-139□  140-159□ 160-189□ >190□ Vet ej □ |
| 1 | Vad är patientens hjärtfrekvens? | <70□ 70-79□ 80-89□ 90-109□ 110-139□  140-159□ 160-189□ >190□ Vet ej □ |
| 1 | Hur många PVK har patienten? | Svar;  Vet ej □ |
| 3 | Vilka undersökningar tror du kommer att behövas utföras närmaste 10 min? | Förslag:  Vet ej □ |
| 2 | Vad är trolig orsak till patientens sepsis? | Förslag:  Vet ej □ |
| 1 | Vad har patienten för halsband? | Svar:  Vet ej □ |
| 3  3  3  3  3 | Hur kommer patientens vitalparametrar utvecklas de 10 närmaste minuterna?  Bedöm utifrån hittills vidtagna åtgärder. | HF= öka□ minska□ oföränd□ vet ej□  BT= öka□ minska□ oföränd□ vet ej□  Sat= öka□ minska□ oföränd□ vet ej□  AF= öka□ minska□ oföränd□ vet ej□  Medvetandet = Stiger □sjunker □ oförändrat □ vet ej□ |

| **Level** | **Fråga** | **Svar** |
| --- | --- | --- |
| 1 | Vad är patientens AF? | <6 □ 6-9 □ 10-15 □ 16-20□ 21-29□ 30-35□ >35□ Vet ej □ |
| 2 | Vilken är bedömningen av C och på vilka grunder? *Svara med kortfattat, scenariet startar snart igen* | Svar:  Vet ej □ |
| 2 | Är patienten tillräckligt syresatt? | Ja □ Nej □ Vet ej □ |
| 1 | Vad är patientens systoliska BT? | <70□ 70-79□ 80-89□ 90-109□ 110-139□  140-159□ 160-189□ >190□ Vet ej □ |
| 3 | Vad har du upplevt blir nästa steg i behandlingen? | Svar:  Vet ej □ |
| 1 | Vad har du fått för uppgifter om patientens tidigare sjukdomar? | Svar;  Vet ej □ |
| 1 | Finns fungerande sug tillgänglig? | Ja □ Nej □  Vet ej □ |
| 3  3  3  3  3 | Hur kommer patientens vitalparametrar utvecklas de 10 närmaste minuterna?  Utifrån hittills vidtagna åtgärder. | HF= öka□ minska□ oföränd□ vet ej□  BT= öka□ minska□ oföränd□ vet ej□  Sat= öka□ minska□ oföränd□ vet ej□  AF= öka□ minska□ oföränd□ vet ej□  Medvetandet = Stiger □sjunker □ oförändrat □ vet ej□ |
